# Difference in spectral power density of sleep EEG between patients with simple snoring and those with obstructive sleep apnoea

**DOI:** 10.1038/s41598-020-62915-x

**Published:** 2020-04-09

**Authors:** Jae Myeong Kang, Seon Tae Kim, Sara Mariani, Seo-Eun Cho, John W. Winkelman, Kee Hyung Park, Seung-Gul Kang

**Affiliations:** 1 Department of Psychiatry, Gil Medical Center, Gachon University College of Medicine, Incheon, Republic of Korea; 2 Department of Otolaryngology, Gil Medical Center, Gachon University College of Medicine, Incheon, Republic of Korea; 3 Division of Sleep & Circadian Disorders, Department of Medicine, Brigham & Women’s Hospital, Harvard Medical School, Boston, MA United States; 4 Department of Neurology, Gil Medical Center, Gachon University College of Medicine, Incheon, Republic of Korea; 5 Departments of Psychiatry and Neurology, Massachusetts General Hospital, Harvard Medical School, Boston, MA United States

**Keywords:** Neuroscience, Neurology

## Abstract

Patients with simple snoring (SS) often complain of poor sleep quality despite a normal apnoea-hypopnoea index (AHI). We aimed to identify the difference in power spectral density of electroencephalography (EEG) between patients with SS and those with obstructive sleep apnoea (OSA). We compared the absolute power spectral density values of standard EEG frequency bands between the SS (*n* = 42) and OSA (*n* = 129) groups during the non-rapid eye movement (NREM) sleep period, after controlling for age and sex. We also analysed partial correlation between AHI and the absolute values of the EEG frequency bands. The absolute power spectral density values in the beta and delta bands were higher in the OSA group than in the SS group. AHI also positively correlated with beta power in the OSA group as well as in the combined group (OSA + SS). In conclusion, higher delta and beta power during NREM sleep were found in the OSA group than in the SS group, and beta power was correlated with AHI. These findings are microstructural characteristics of sleep-related breathing disorders.

## Introduction

Obstructive sleep apnoea (OSA) is the most prevalent manifestation of sleep disordered breathing (SDB) characterised by repeated episodes of complete or partial collapse of upper airway during sleep^1^. Clinical manifestation of OSA includes snoring, disturbed sleep, fatigue, daytime sleepiness, loss of concentration, memory decline, and neuropsychiatric symptoms^2–5^. OSA is diagnosed using overnight polysomnography (PSG) when the apnoea or hypopnoea occurs five or more times per hour (apnoea-hypopnoea index [AHI] ≥ 5)^6^. In the spectrum of SDB, snorers with AHI < 5 are defined as having simple snoring (SS), which has less pathological and clinical significance^7–9^.

SS is a preclinical condition that requires no treatment. However, patients with SS may suffer from daytime sleepiness, insomnia, and psychiatric symptoms despite a normal AHI^10,11^. Although SS is differentiated from OSA by AHI on PSG, there is a weak association between AHI and symptomatology in SDB^12–18^. Some studies reported that patients with SS have more severe psychiatric symptoms and poorer subjective sleep quality than those with OSA^12,19^. This paradoxical association between AHI, subjective sleep quality, and psychiatric symptoms suggests that other factors besides AHI influence such symptoms, and it may be necessary to study differences in microstructure in addition to classical PSG measurements (e.g., sleep stage ratio or sleep efficiency) and macrostructures.

Researchers have previously attempted to investigate the microstructure of PSG-derived sleep as well as waking quantitative electroencephalography (qEEG) in SDB^20–24^. It has been generally accepted that wake EEG slowing is more pronounced in patients with OSA than in good sleepers, and the slowing is associated with daytime sleepiness and attention or vigilance dysfunction^25–31^. Studies using sleep EEG derived from PSG have documented significant differences in spectral power or sleep spindles during non-rapid eye movement (NREM) sleep between patients with OSA and controls^32–35^. Although previous spectral analysis studies have investigated EEG and oximetric spectral features to improve OSA detection^21^, little is known about differences in sleep qEEG between patients with SS and those with OSA.

Our goal was to study differences in qEEG between SS and OSA to understand the association between qEEG and AHI in SDB. The aims of this study were 1) to compare the power spectral density of qEEG frequency bands during NREM sleep between the SS and OSA groups and 2) to investigate the correlation between AHI and qEEG power in patients with SDB.

## Results

### Demographic and PSG characteristics of participants

Among the 171 participants, 129 (75.4%) were classified into the OSA group and 42 (24.6%) were classified into the SS group. Demographic and PSG characteristics of participants are presented in Table 1. There were no significant differences between the groups in terms of age and sex. As expected, the groups differed in body mass index (BMI), AHI, and arousal index and all values were higher in the OSA group than in the SS group. PSG data differed in the ratio of sleep stages N1 and N2 but did not differ in most other PSG measures (the ratio of stage N3 and REM and all sleep and wake time data, including total sleep time and sleep efficiency) between the groups.

**Table 1:** Demographic, clinical, and polysomnographic characteristics of subjects.

Variable	SS (*n* = 42)	OSA (*n* = 129)	Statistics^‡^
**Demographics**			
Age, years	44.2 ± 12.4	46.4 ± 11.0	*t* = –1.04, *p* = 0.299
Sex, male	34 (81.0%)	110 (85.3%)	χ^2^ = 0.44, *p* = 0.505
Body mass index, kg/m^2^	24.2 ± 2.9	26.2 ± 3.5	*t* = –3.36, *p* = 0.001
**Polysomnographic data**			
**Sleep and wake time**			
Time in bed, min	416.8 ± 26.3	410.7 ± 30.0	*t* = 1.19, *p* = 0.237
Total sleep time, min	340.2 ± 57.0	337.8 ± 55.8	*t* = 0.24, *p* = 0.809
Sleep latency, min	13.4 ± 19.5	13.4 ± 22.6	*t* = 0.00, *p* = 0.996
Sleep efficiency, %	81.9 ± 14.5	82.2 ± 13.3	*t* = –0.11, *p* = 0.914
WASO, min	63.3 ± 58.7	58.8 ± 45.0	*t* = 0.55, *p* = 0.584
REM sleep latency, min	132.5 ± 71.4	124.3 ± 62.6	*t* = 0.72, *p* = 0.475
**Sleep stage, %**			
N1	18.1 ± 9.1	30.5 ± 16.3	*t* = –6.18, *p* < 0.001
N2	61.3 ± 8.9	50.3 ± 14.8	*t* = 5.82, *p* < 0.001
N3	4.3 ± 6.3	3.1 ± 5.3	*t* = 0.13, *p* = 0.202
R	16.3 ± 7.0	15.6 ± 7.2	*t* = 0.66, *p* = 0.596
**Respiration**			
AHI, event per hour	1.8 ± 1.4	31.7 ± 21.2	*t* = –15.92, *p* < 0.001
Arousal index	17.76 ± 8.36	35.47 ± 17.98	*t* = –8.67, *p* < 0.001

Data are mean ± standard deviation or *n* (%) values. ^‡^Independent *t* test *or χ*^2^ test; SS, simple snoring; OSA, obstructive sleep apnoea; WASO, wake time after sleep onset; REM, rapid eye movement; N1, non-REM (NREM) stage 1; N2, NREM stage 2; N3, NREM stage 3; R, REM stage; AHI, apnoea hypopnoea index.

### Comparison of absolute spectral EEG power between groups

**Figure 1:**
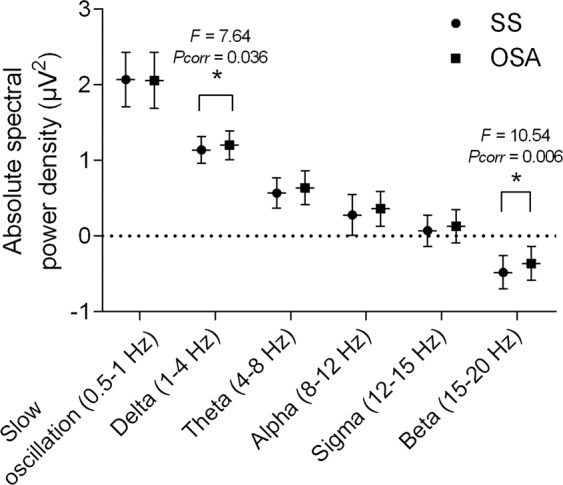
Comparisons of the absolute power spectral density during NREM sleep between the SS and OSA groups. *p < 0.05 in ANCOVA controlling for age and sex after Bonferroni correction (uncorrected *p* value × 6) for correction of multiple comparisons. Bars denote mean ± standard deviation. NREM, non-rapid eye movement; SS, simple snoring; OSA, obstructive sleep apnoea; ANCOVA, analysis of covariance.

Table 2 and Fig. 1 provide the comparison of the absolute spectral power in central electrodes during NREM sleep between the SS and OSA groups. There were significant differences in the spectral power in the delta and beta frequency bands between the groups after controlling for age and sex. The absolute spectral power in the delta (1–4 Hz, *F* = 10.54, *p* = 0.001, *p* corrected = 0.006) and beta (15–20 Hz, *F* = 7.64, *p* = 0.006, *p* corrected = 0.036) bands during NREM sleep was higher in the OSA group than in the SS group. Comparative results during N2, N3 and REM sleep are presented in Table S1 in the supplementary information.

**Table 2:** Comparison of the absolute spectral power density^§^ during NREM sleep between SS and OSA groups after controlling age and sex.

Spectral bands	SS (n = 42)	OSA (n = 129)	Statistics* (ANCOVA)
Slow oscillation (0.5–1 Hz)	2.07 ± 0.36	2.06 ± 0.37	*F* = 0.32, *p* = 0.572, *p corr* > 0.999
Delta (1–4 Hz)	1.14 ± 0.18	1.20 ± 0.19	*F* = 7.64, *p* = 0.006, ***p corr*** = **0**.**036**
Theta (4–8 Hz)	0.57 ± 0.20	0.64 ± 0.22	*F* = 6.30, *p* = 0.013, *p corr* = 0.078
Alpha (8–12 Hz)	0.28 ± 0.27	0.36 ± 0.23	*F* = 4.48, *p* = 0.036, *p corr* = 0.216
Sigma (12–15 Hz)	0.07 ± 0.21	0.13 ± 0.22	*F* = 3.19, *p* = 0.076, *p corr* = 0.456
Beta (15–20 Hz)	−0.48 ± 0.22	−0.36 ± 0.22	*F* = 10.54, *p* = 0.001, ***p corr*** = **0**.**006**

Data are mean ± standard deviation. ^**§**^log-transformed spectral power density (log_10_ μV^2^); EEG, electroencephalography; NREM, non-rapid eye movement; SS, simple snoring; OSA, obstructive sleep apnoea; *Controlling for age and sex; ANCOVA, analysis of covariance; *p corr*, *p* value after Bonferroni correction (uncorrected *p* value × 6) for correction of multiple comparisons. The number in bold indicates significance after Bonferroni correction (*p* < 0.05).

Table 3 presents results for comparisons of the absolute spectral power in central electrodes among SS, mild OSA (5 ≤ AHI < 15), moderate OSA (15 ≤ AHI < 30), and severe OSA (30 ≤ AHI) groups to reveal differences in the EEG power according to the severity of AHI. There were significant differences in the spectral power of the beta frequency bands between the groups after controlling for age and sex. The absolute spectral power of the beta (*F* = 4.41, *p* = 0.005, *p* corrected = 0.031) band during NREM sleep was higher in the severe OSA group than in the SS group following post-hoc analysis.

**Table 3:** Comparison of the absolute spectral power density^§^ during NREM sleep among SS, mild OSA, moderate OSA, and severe OSA groups after controlling age and sex.

Spectral bands	SS (*n* = 42)	Mild OSA (*n* = 36)	Moderate OSA (*n* = 29)	Severe OSA (*n* = 64)	Statistics * (ANCOVA)
Slow oscillation (0.5–1 Hz)	2.07 ± 0.36	2.05 ± 0.35	2.02 ± 0.28	2.10 ± 0.40	*F* = 0.30, *p* = 0.827, *p corr* > 0.999
Delta (1–4 Hz)	1.14 ± 0.18	1.20 ± 0.21	1.20 ± 0.22	1.20 ± 0.18	*F* = 2.57, *p* = 0.056, *p corr* = 0.336
Theta (4–8 Hz)	0.57 ± 0.20	0.65 ± 0.26	0.68 ± 0.22	0.62 ± 0.20	*F* = 2.20, *p* = 0.090, *p corr* = 0.539
Alpha (8–12 Hz)	0.28 ± 0.27	0.39 ± 0.26	0.38 ± 0.22	0.32 ± 0.21	*F* = 1.73, *p* = 0.163, *p corr* = 0.978
Sigma (12–15 Hz)	0.07 ± 0.21	0.14 ± 0.24	0.11 ± 0.19	0.13 ± 0.22	*F* = 1.59, *p* = 0.193, *p corr* > 0.999
Beta (15–20 Hz)	−0.48 ± 0.22	−0.38 ± 0.24	−0.37 ± 0.21	−0.34 ± 0.22	*F* = 4.41, *p* = 0.005, ***p corr*** = **0**.**031**

Data are mean ± standard deviation. ^**§**^log-transformed spectral power density (log_10_ μV^2^); EEG, electroencephalography; NREM, non-rapid eye movement; SS, simple snoring; OSA, obstructive sleep apnoea; *Controlling for age and sex; ANCOVA, analysis of covariance; *p corr*, *p* value after Bonferroni correction (uncorrected *p* value × 6) for correction of multiple comparisons. The number in bold indicates significance after Bonferroni correction (*p* < 0.05).

### Partial correlation analyses between AHI, arousal index and absolute power spectral density

**Figure 2:**
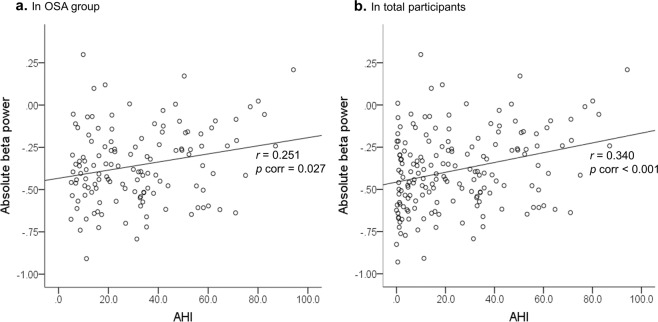
Partial correlations between AHI and absolute beta power during NREM sleep. (**a**) Partial correlation between AHI and absolute beta power during NREM period controlling for age and sex in the OSA group (*r* = 0.251, *p* corr = 0.027). (**b**) Partial correlation between AHI and absolute beta power during NREM period controlling for age and sex in total participants (*r* = 0.340, *p* corr <0.001). AHI, apnoea-hypopnea index; NREM, non-rapid eye movement; OSA, obstructive sleep apnoea; *r*, Pearson’s r; Log transformation for spectral EEG was performed before Pearson correlation analysis when both variables deviate from the normal distribution. *p* corr, *p* value after Bonferroni correction (uncorrected *p* value × 6) for correction of multiple comparisons.

Table 4 presents partial correlation analyses results between AHI and absolute power spectral density after controlling for age and sex in the SS, OSA, and total participants. AHI positively correlated with absolute beta power in the OSA group (*r* = 0.251, *p* = 0.004, *p* corrected = 0.027; Fig. 2a) and total participants (SS + OSA; *r* = 0.340, *p* < 0.001, *p* corrected <0.001; Fig. 2b).

**Table 4:** Partial correlation between AHI and absolute spectral power density during NREM sleep after controlling for age and sex.

Variables	Total (*n* = 171)			SS (*n* = 42)			OSA (*n* = 129)		
	r*	*p* value	*p corr*	r*	*p* value	*p corr*	r*	*p* value	*p corr*
Slow oscillation	0.101	0.192	>0.999	0.036	0.826	>0.999	0.098	0.274	>0.999
Delta	0.151	0.051	0.360	0.035	0.830	0.906	0.026	0.769	>0.999
Theta	0.126	0.103	0.618	0.146	0.369	>0.999	−0.016	0.861	>0.999
Alpha	0.090	0.247	0.024	0.039	0.812	>0.999	−0.044	0.629	>0.999
Sigma	0.161	0.036	0.216	−0.170	0.295	>0.999	0.087	0.334	>0.999
Beta	0.340	<0.001	**<0**.**001**	−0.071	0.663	>0.999	0.251	0.004	**0**.**027**

AHI, apnoea-hypopnea index; EEG, electroencephalography; NREM, non-rapid eye movement; SS, simple snoring; OSA, obstructive sleep apnoea; *r*, Pearson’s *r*. *Log transformation for spectral EEG was performed before Pearson correlation analysis when both variables deviate from normal distribution. *p* corr, *p* value after Bonferroni correction (uncorrected *p* value × 6) for correction of multiple comparisons. The number in bold indicates significance after Bonferroni correction (*p* < 0.05).

Table S2 in supplementary information presents partial correlation analyses results between the arousal index and absolute power spectral density after controlling for age and sex in the SS, OSA and total participants. The arousal index positively correlated with absolute beta power in all participants (SS + OSA; *r* = 0.282, *p* < 0.001, *p* corrected <0.001; Fig. S1).

## Discussion

We conducted spectral power analysis of sleep EEG derived from PSG in patients with suspected OSA. The major finding of the present study is the spectral power difference in the delta and beta frequency bands of sleep EEG between the SS and OSA groups (Table 2). We found increased delta and beta activity^36^ in the OSA group compared to the SS group. In addition, the beta power positively correlated with AHI in the OSA group and total participants.

We attribute this seemingly paradoxical result—an increase in delta activity, which predominates in slow wave sleep, in patients with higher AHI—as (1) a compensatory EEG slowing response to arousal or (2) precedence of slow waves over respiratory arousal in patients with OSA. Studies on the hypothesis of compensatory EEG slowing response have been previously published^37,38^. Previous studies reported that the delta band amplitude increased in patients with OSA starting few seconds after apnoea onset during NREM sleep^38^. Another study reported that consecutive respiratory events restoring process was related to higher spectral power of the delta band^37^. These findings may implicate that the slowing of EEG in OSA and slow waves, including the delta band, play a role in recovery from altered respiration and complement arousal event during sleep. On the other hand, there is an opinion that the increase of delta is not compensating for arousal due to respiratory disturbance, but more hypopnea related arousal response occurs after slow wave sleep in OSA patients. Previous studies have shown that arousals in NREM sleep were consistently preceded by increase in the transient delta power in both normal and pathological NREM sleep^39^.

The lower delta power in the SS group than in the OSA group may be the underlying cause of subjective symptoms in patients with SS or subjective-objective mismatch in sleep perception in SDB, as shown in previous studies^10–13^. In other words, reduced delta power in the SS group may induce poor sleep perception and eventually develop poor psychiatric symptoms. Although the severity of SDB continuum is defined as mild form of SS to severe form of OSA^9^, a weak or no relationship between AHI and accompanying psychiatric symptoms, such as depression and anxiety, has been reported^16^. We also found increased psychiatric symptoms^12^ and perception of nocturnal SDB symptoms^40^ in the SS group compared to those in the OSA group. Bianchi *et al*. reported subjective-objective mismatch in sleep perception in patients with sleep apnoea and insomnia and concluded that mismatch was not attributable to commonly measured PSG measures and that further insight into the advanced signal processing technique would help explain this mismatch^41^. Thus, further elaborate investigations into the relationship among sleep EEG spectral power, sleep perception, and psychiatric symptoms in SDB would be necessary.

We found higher beta power in the OSA group than in the SS group (Table 2) and the absolute beta power density positively correlated with the AHI and arousal index in all participants (Table 4 and Table S2). Beta band is associated with emotional and cognitive processes and arousal during sleep^42^ and is enhanced during the N1 and N2 sleep stages and decreased during deeper sleep^36^. Higher beta power in patients with OSA in our study can be explained by the higher background brain activity in patients with OSA than in those with SS. This study also showed a significant correlation between the arousal index and beta power, which means sleep disturbance of SDB. Positive correlation between beta power in NREM sleep and AHI is consistent with recent data^24^. Our result replicated this data and had the same association in the suspected OSA (SS + OSA groups). In addition, we believed that the increase in both beta and delta powers during NREM sleep in the OSA group was due to cyclic alternating patterns, which are considered to be indicators of arousal instability^43^. The A phase in the cyclic alternating patterns was reported to be associated with increase in beta power and preceding delta power^39^. Our results exhibiting positive correlation between beta power and the arousal index in NREM sleep may also support this cyclic alternating pattern.

Our study was limited by the small sample size of the SS group. Further, the sample sizes between the SS and OSA groups were unequal owing to the clinical characteristics of the participants. However, as this is the largest investigation of spectral power comparison between the SS and OSA groups to date, this study is meaningful. Future studies with a larger sample size are needed involving patients with SS and OSA as well as normal controls.

## Conclusions

In summary, we found that qEEG spectral power in the delta and beta bands of NREM sleep differ between the SS and OSA groups. The higher delta and beta power were found in the OSA group than SS group and beta power was correlated with AHI in combined group. This study reports differences in microstructures of PSG-derived sleep EEG between SS and OSA and how these differences in microstructures may reveal areas that were not previously explained by macrostructure and traditional parameters in PSG. The current finding of the increased delta power in the OSA group might explain previous reports on the lack of an association between the AHI and subjective symptoms in patients with SDB^10–13^. Future investigations need to focus on the characterisation of features of SDB in both conventional and microstructural aspects of PSG in order to clarify the neural mechanism of SDB and explain clinical phenomena not explained by conventional PSG.

## Methods

### Participants

Adults (age: 18–65 years) with suspected OSA were recruited from the sleep clinic of Gil Medical Center from March 2012 to February 2016. Among 205 participants who were recruited through structured clinical interviews and screening scales, 171 participants were finally included in the analyses. All participants had symptoms of SS or OSA, such as frequent snoring, witnessed apnoea, experience of choking during sleep, and daytime sleepiness. They met the breathing-related sleep disorder diagnostic criteria of the Diagnostic and Statistical Manual of Mental Disorders (fourth edition, text revision)^44^. Subjects were interviewed and evaluated by medical doctors (i.e., board-certified medical doctors in the department of psychiatry, neurology, and Otolaryngology) with over 5 years of experience in OSA and sleep medicine.

The exclusion criteria for participants were as follows: (i) comorbidity of severe medical or surgical conditions; (ii) major psychiatric disorders; (iii) previous diagnosis with OSA; (iv) history of uvulopalatopharyngoplasty; and (v) suspected as having other major sleep disorders such as restless legs syndrome, REM sleep behaviour disorder, circadian rhythm sleep disorder, or narcolepsy.

Written informed consent was obtained from all participants prior to inclusion in the study, and the Institutional Review Board of Gil Medical Center approved all study protocols. All experiments were performed in accordance with relevant guidelines and regulations.

### Polysomnography

In-laboratory and monitored nocturnal PSG were conducted for all participants. Standard PSG recordings included six electroencephalogram leads (F3, F4, C3, C4, O1, and O2), one electrocardiography channel, two electrooculogram channels (E1-M2 and E2-M2), and three electromyography channels (chin and both anterior tibialis muscles) and were conducted according to the recommendations of American Academy of Sleep Medicine (AASM)^45^. PSG was conducted using COMET systems (Grass-Telefactor Corporation), and PSG results were scored based on the criteria in the AASM manual^45^. We used the recommended rules of hypopnea (≥30% reduction in nasal pressure signal excursions from baseline that lasted ≥10 s and were associated with ≥4% desaturation from the pre-event baseline) during sleep in the AASM manual^45^ and OSA was defined as an AHI ≥ 5 and SS as AHI < 5. Experienced PSG technologists who completed the interscorer reliability program of the AASM (http://www.aasmnet.org/isr/) scored PSG recordings using the AASM sleep scoring criteria^45^. All PSG data were confirmed by a sleep specialist medical doctor (K.H.P.).

### Spectral analysis

Power spectra were computed for each EEG frequency band: slow oscillation (0.5–1 Hz), delta (1–4 Hz), theta (4–8 Hz), alpha (8–12 Hz), sigma (12–15 Hz), and beta (15–20 Hz). For the present analysis, data derived from stages N2, N3, REM and the average NREM central EEG electrode (i.e., [C3/A2 + C4/A1]/2) were used.

SpectralTrainFig was used for power spectral analysis. This program is an automated open-source Matlab graphic interface for the spectral analysis of sleep EEG in PSG. It detects and deletes epochs with artifact automatically and generates summary figures for visual adjudication^46,47^. It was developed by the National Sleep Research Resource (https://github.com/nsrr/SpectralTrainFig)^48,49^. In accordance with Welch’s method, the spectral power density was calculated using 10 overlapping 4-s sub-epochs for each 30-s epoch, with a 50% tapered cosine window. The artifact due to electrocardiogram interference was removed using a template subtraction method^50^. Manual visual adjudication was performed by a researcher who was blinded to the subject group, and spectra data with significant artifacts were excluded manually. In spectral analysis using the SpectralTrainFig program and visual adjudication, 34 subjects were excluded due to excessive PSG artifacts.

### Statistical analysis

G*Power 3.1.9.2 software was used for sample size calculation. The minimum sample size yielded from this program was 134 (34 for group 1 and 100 for group 2). In the power analysis, a medium effect size of 0.5, power of 0.8 and α error probability of 0.05 were applied with group allocation ratio of 3 (OSA:SS = 3:1) based on the previous works^12,13^.

IBM SPSS for Windows software (version 23.0, IBM Corp, Armonk, NY, USA) was used for data analysis. Chi-square test was used for categorical variables, and independent t-test analysis of variance or analysis of covariance was used to compare the demography, PSG characteristics, and absolute spectral EEG power between the groups. Pearson correlation analysis was used to assess the correlation between AHI, arousal index and absolute spectral EEG power. Log transformation of spectral EEG power was conducted before Pearson correlation analysis when both variables showed deviation from normal distribution. P < 0.05 after Bonferroni correction was considered statistically significant.

## Supplementary information

Supplementary Information.
