# Supplementary material for: Difference in spectral power density of sleep EEG between patients with simple snoring and those with obstructive sleep apnoea
Source: Sci Rep. 2020 Apr 9;10:6135. doi: 10.1038/s41598-020-62915-x (PMC7145832; doi:10.1038/s41598-020-62915-x)
Supplement: Supplementary file 1 — Supplementary Information. [file 41598_2020_62915_MOESM1_ESM.docx]

**Difference in spectral power density of sleep EEG between patients with simple snoring and those with obstructive sleep apnoea**

Jae Myeong Kang ^a,*^ , Seon Tae Kim ^b,*^, Sara Mariani ^c^, Seo-Eun Cho ^a^

John W. Winkelman ^e^, Kee Hyung Park ^d,†^, Seung-Gul Kang ^a,††^

*^a^ Department of Psychiatry, Gil Medical Center, Gachon University College of Medicine, Incheon, Republic of Korea*

*^b^Department of* *Otolaryngology, Gil Medical Center, Gachon University College of Medicine, Incheon, Republic of Korea*

*^c^Division of Sleep & Circadian Disorders, Department of Medicine, Brigham & Women's Hospital, Harvard Medical School, Boston, MA, United States*

*^d^Department of Neurology, Gil Medical Center, Gachon University College of Medicine, Incheon, Republic of Korea*

*^e^ Departments of Psychiatry and Neurology, Massachusetts General Hospital, Harvard Medical School, Boston, MA, United States.*

| **Table S1.** Comparison of the absolute spectral power density^§^ during stage N2, N3, and R between SS and OSA groups after controlling age and sex | | | |
| --- | --- | --- | --- |
| *Spectral bands* | *SS (n=42)* | *OSA (n=129)* | *Statistics** *(ANCOVA)* |
| In N2 sleep |  |  |  |
| Slow oscillation (0.5-1 Hz) | 2.11±0.36 | 2.14±0.35 | *F*=1.17, *p*=0.282, *p corr*>0.999 |
| Delta (1-4 Hz) | 1.18±0.17 | 1.28±0.17 | *F*=15.54, *p<*0.001, ***p corr<*0.001** |
| Theta (4-8 Hz) | 0.60±0.20 | 0.68±0.21 | *F*=8.92, *p*=0.003, ***p corr*=0.018** |
| Alpha (8-12 Hz) | 0.29±0.27 | 0.22±0.02 | *F*=5.27, *p*=0.023, *p corr*=0.138 |
| Sigma (12-15 Hz) | 0.11±0.22 | 0.18±0.22 | *F*=3.33, *p*=0.070, *p corr*=0.420 |
| Beta (15-20 Hz) | –0.50±0.22 | –0.39±0.24 | *F*=7.85, *p*=0.006, ***p corr*=0.036** |
| In N3 sleep |  |  |  |
| Slow oscillation (0.5-1 Hz) | 2.75±0.46 | 2.77±0.39 | *F*=0.93, *p*=0.338, *p corr*>0.999 |
| Delta (1-4 Hz) | 1.54±0.21 | 1.64±0.19 | *F*=6.55, *p*=0.012, *p corr*=0.072 |
| Theta (4-8 Hz) | 0.72±0.20 | 0.86±0.23 | *F*=10.40, *p*=0.002, ***p corr*=0.012** |
| Alpha (8-12 Hz) | 0.26±0.30 | 0.43±0.27 | *F*=8.65, *p*=0.004, ***p corr*=0.024** |
| Sigma (12-15 Hz) | –0.09±0.21 | 0.09±0.24 | *F*=15.81, *p<*0.001, ***p corr<*0.001** |
| Beta (15-20 Hz) | –0.70±0.19 | –0.48±0.28 | *F*=17.44, *p*<0.001, ***p corr<*0.001** |
| In REM sleep |  |  |  |
| Slow oscillation (0.5-1 Hz) | 1.55±0.34 | 1.65±0.34 | *F*=4.75, *p*=0.031, *p corr*=0.186 |
| Delta (1-4 Hz) | 0.77±0.17 | 0.81±0.19 | *F*=2.75, *p*=0.099, *p corr*=0.594 |
| Theta (4-8 Hz) | 0.34±0.20 | 0.38±0.22 | *F*=0.77, *p*=0.381, *p corr*>0.999 |
| Alpha (8-12 Hz) | 0.003±0.26 | 0.09±0.26 | *F*=4.70, *p*=0.032, *p corr*=0.192 |
| Sigma (12-15 Hz) | –0.35±0.27 | –0.25±0.25 | *F*=5.64, *p=*0.019, *p corr=*0.114 |
| Beta (15-20 Hz) | –0.50±0.27 | –0.40±0.26 | *F*=5.02, *p*=0.026, *p corr=*0.156 |
| Data are mean±SD; **^§^**log-transformed spectral power density (log_10_ μV^2^); EEG, electroencephalography; SS, simple snoring; OSA, obstructive sleep apnoea; *Controlling for age and sex; ANCOVA, analysis of covariance; REM, rapid eye movement; *p corr*, *p* value after Bonferroni correction (uncorrected *p* value × 6) for correction of multiple comparisons. The number in bold indicates significance after Bonferroni correction (*p* < 0.05) | | | |

| **Table S2.** Partial correlation between the arousal index and absolute spectral power density during NREM sleep after controlling for age and sex | | | | | | | | | | | |
| --- | --- | --- | --- | --- | --- | --- | --- | --- | --- | --- | --- |
| *Variables* | Total (*n*=171) | | |  | SS (*n*=42) | | |  | OSA (*n*=129) | | |
|  | *r* | *p value* | *p corr* |  | *r* | *p value* | *p corr* |  | *r* | *p value* | *p corr* |
| Slow oscillation | 0.011 | 0.891 | 0.066 |  | 0.100 | 0.539 | >0.999 |  | –0.039 | 0.663 | >0.999 |
| Delta | –0.013 | 0.863 | >0.999 |  | -0.038 | 0.814 | 0.906 |  | –0.128 | 0.152 | 0.912 |
| Theta | 0.024 | 0.759 | >0.999 |  | 0.079 | 0.628 | >0.999 |  | –0.104 | 0.246 | >0.999 |
| Alpha | 0.063 | 0.419 | >0.999 |  | 0.040 | 0.808 | >0.999 |  | –0.032 | 0.717 | >0.999 |
| Sigma | 0.092 | 0.236 | >0.999 |  | 0.104 | 0.523 | >0.999 |  | 0.022 | 0.806 | >0.999 |
| Beta | 0.282 | <0.001 | **<0.001** |  | 0.232 | 0.150 | 0.900 |  | 0.202 | 0.023 | 0.162 |
| NREM, non-rapid eye movement; SS, simple snoring; OSA, obstructive sleep apnoea; *r*, Pearson’s *r*; *p* corr, *p* value after Bonferroni correction (uncorrected *p* value × 6) for correction of multiple comparisons. The number in bold indicates significance after Bonferroni correction (*p* < 0.05) | | | | | | | | | | | |

**Figure S1**. Partial correlations between the arousal index and absolute beta power during NREM sleep all total participants


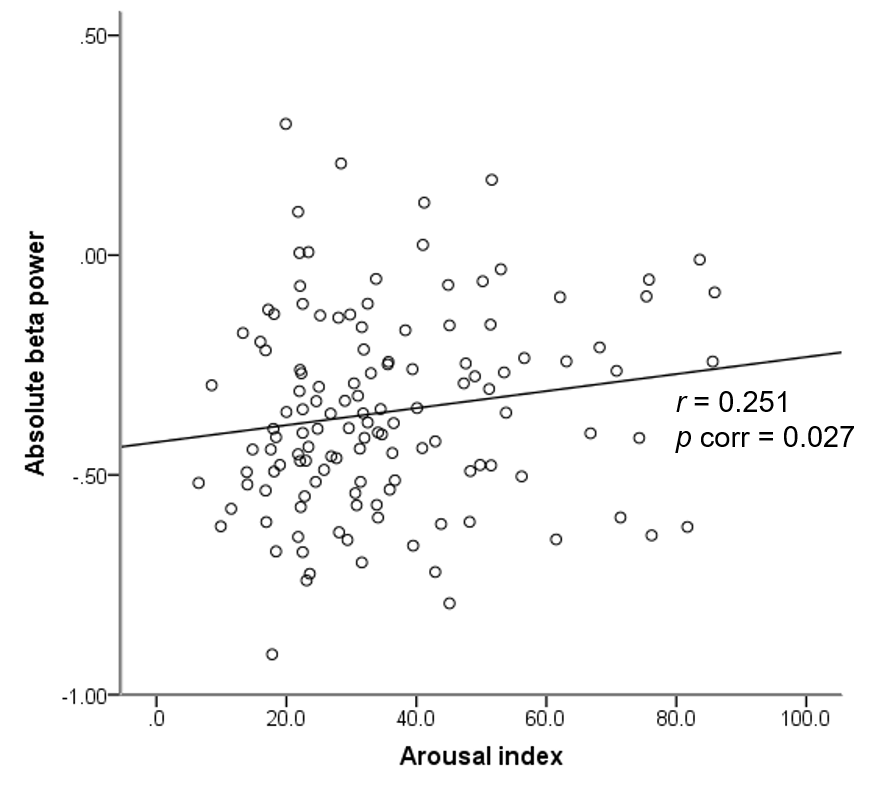


Figure S1 legend: NREM, non-rapid eye movement; *r*, Pearson’s r; *p* corr, *p* value after Bonferroni correction (uncorrected *p* value × 6) for correction of multiple comparisons.
